# Effect of sarcopenia on risk of atrial fibrillation: a systematic review and meta-analysis of observational studies

**DOI:** 10.3389/fcvm.2026.1721219

**Published:** 2026-04-01

**Authors:** Jie Liu, Zhen Li, Yanhong Bi, Xiaoyu Che, Ao Feng, Yazhuo Liu, Yiou Wang, Simiao Tian

**Affiliations:** 1 Department of Clinical Nutrition, Affiliated Zhongshan Hospital of Dalian University, Dalian, China; 2 Department of Medical Records and Statistics, Affiliated Zhongshan Hospital of Dalian University, Dalian, China; 3 Department of Research, Affiliated Zhongshan Hospital of Dalian University, Dalian, China; 4 Department of Prevention and Healthcare, Affiliated Zhongshan Hospital of Dalian University, Dalian, China

**Keywords:** atrial fibrillation, meta-analysis, prospective study, sarcopenia, skeletal muscle index

## Abstract

**Background:**

The association between sarcopenia and risk of atrial fibrillation (AF) remains uncertain. In this study, we aimed to quantify the association between sarcopenia and the risk of AF through a meta-analysis of observational cohort studies.

**Methods:**

PubMed, Web of Science, EMBASE, and major scientific conference sessions were searched without language restrictions from inception to 15 August 2025. Bibliographies of relevant articles were also manually searched. Observational studies that evaluated the association between sole or combined components of sarcopenia criteria and risk of AF were included.

**Results:**

Ten studies involving 7,358,442 participants were included in the meta-analysis. Sarcopenia was significantly associated with an increased risk of AF onset [pooled hazard ratio (HR) = 1.44, 95% CI: 1.22–1.68], with high heterogeneity among studies (*I*^2^ = 93.5%, *P* < 0.001) but no indication of publication bias (Begg's *P* = 0.728 and Egger's *P* = 0278). This association remained significant by sole or concurrent presence of low muscle mass and impaired muscle function, with HR of 1.26 (95% CI: 1.05–1.50) for only impaired muscle function used and HR of 1.79 (95% CI: 1.49–2.15) for comprehensive criteria with two combined components. Subgroup analyses showed that the risk of AF did not appreciably change across sex, age (<60 and ≥60 years), body mass index (BMI) status (non-obese or obese), physical activity (yes or no), duration of follow-up (≥10 or <10 years), and geography (Asian cohorts or non-Asian cohorts), with HRs ranging from 1.22 in young participants with sarcopenia to 1.97 in women with sarcopenia.

**Conclusions:**

The results of our study demonstrated that sarcopenia is associated with a 44% increased risk of AF onset compared with non-sarcopenia individuals. The association was independent of sex, age, geographic location, duration of follow-up, BMI status, and regular physical activity.

**Systematic Review Registration:**

https://www.crd.york.ac.uk/PROSPERO/view/CRD420251020653 identifier, CRD420251020653.

## 1 Introduction

Atrial fibrillation (AF) is the most common serious cardiac arrhythmia worldwide, with growing prevalence, particularly among the elderly (1). Due to population aging and the high presence of AF-related risk factors, the burden of AF is projected to reach 15.9 million adults in the US by 2050 (2) and 17.9 million adults in Europe by 2060 (3), with more than half of these cases occurring in the elderly aged ≥80 years (4). Despite improved pathophysiological understanding and significant progress in treatment, AF is still associated with multiple adverse health outcomes, such as stroke, heart failure, cognitive impairment, and sudden cardiac arrest (5), leading to an increased risk of mortality and morbidity, and a global loss of 6.0 million disability-adjusted life-years (6).

Sarcopenia, a geriatric syndrome characterized by the generalized loss of muscle mass and muscle function with aging, is recognized as a common but understudied complication in patients with AF, affecting up to 16.3% of patients who suffer from sarcopenia (7). Recent data from a meta-analysis reported that estimated incidence of sarcopenia ranged from 10% to 27% according to sociodemographic characteristics and different geographic regions (8). Sarcopenia is shown to be associated with multiple adverse outcomes, including falls, physical frailty, fractures, deficient quality of life, hospitalization, and mortality (9–11). Thus, it is becoming a major health concern for aging societies at both the patient and the societal levels and is associated with considerable healthcare costs (12).

Recent evidence has expanded our understanding of sarcopenia’s effect on cardiovascular diseases (CVD), including heart failure (13), valvular heart disease (14), atherosclerosis (15), and peripheral arterial disease (16) (17). Of particular interest is the link of sarcopenia to AF, since these two share common physiological and endocrine mechanisms, as well as exogenous mechanisms such as physical inactivity, which exacerbate both conditions (18, 19). A prospective study reported that sarcopenia was independently associated with a 2-fold increase in AF risk after a 10-year follow-up (20), whereas another study with a short-term follow-up revealed a contrasting finding with non-significant association (21). The association between sarcopenia and AF remains inconclusive due to multifactorial reasons, and to date, few meta-analyses have systematically evaluated the impact of sarcopenia on AF risk. Therefore, the aim of this study was to clarify the association of sarcopenia with AF risk by performing a meta-analysis of related published prospective studies.

## 2 Materials and methods

### 2.1 Protocol and registration

This systematic review and meta-analysis was conducted in accordance with the Cochrane Handbook for Systematic Reviews (22). This meta-analysis was performed in compliance with the updated PRISMA (2020) (23) and the Meta-analysis of Observational Studies in Epidemiology guidelines (24). The study protocol was prospectively registered in the International Prospective Register of Systematic Reviews (PROSPERO, ID: CRD420251020653).

### 2.2 Literature search strategy

We systematically searched PubMed, Web of Science, and Embase using appropriate search terms from inception up to 15 August 2025, without language restrictions, to identify relevant full-text studies which examined the association between sarcopenia or muscle mass and risk of AF in adults. A manual search was also performed of these electronic database and reference lists.

The key search terms included sarcopenia, muscle mass, muscle function, and AF. We restricted the search to human and full-text studies only. Two investigators—JL and ZL—independently completed the title/abstract screening for eligibility using a preplanned list of inclusion/exclusion criteria, with discrepancies resolved by consensus or discussion with a third investigator (either ST or YW).

### 2.3 Study selection and inclusion criteria

The retrieved studies were considered for inclusion if they met the following criteria: (1) observational studies of cohort, cross-sectional or case–control design that focused on the relationship between sarcopenia and risk of AF in adults; (2) diagnosis of sarcopenia based on consensus criteria—such as the European Working Group on Sarcopenia in Older People (EWGSOP) and the Asian Working Group for Sarcopenia (AWGS)—or study-reported definition that applied at least one of the three following aspects: low muscle mass, low muscle strength, and/or low physical performance; (3) low lean skeletal muscle mass detected using instruments commonly used in previous studies, such as dual-energy X-ray absorptiometry (DXA), bioelectrical impedance analysis (BIA), magnetic resonance imaging, and computed tomography; (4) the age of the included subjects to be ≥18 years; and (5) reporting of hazard ratio (HR), risk ratio (RR), or odds ratio (OR) with the corresponding 95% confidence interval (95% CI). Studies were excluded if they (1) did not report study outcomes or (2) were duplicate publications. Titles and abstracts were screened initially by two independent investigators (JL and ZL), followed by full-text review of relevant articles in accordance with the inclusion and exclusion criteria. For studies with duplicate cohorts, we included the study with the most recent data, larger sample size, and/or more available data for subgroup analyses. Any discrepancy was resolved by consensus or discussion with the third investigator (ST or YW).

### 2.4 Data extraction

Two investigators (JL and YB) independently extracted the following data in a standardized format from each eligible study: the first author's name, year of publication, study design, study location, data sources (general or specific cohort), duration of follow-up, sample size, age range at baseline year, sex distribution, sarcopenia definition, methods of measuring muscle mass or muscle function, adjustments, and relevant outcomes of interest (i.e., effect size represented by HR, RR, and OR). If multiple sarcopenia definitions were used in one study, we treated each definition as an independent one. If relevant data were not readily accessible, authors were contacted to obtain the supplemental data. All disagreements were settled by discussion with a third investigator (XC) till a consensus was reached.

### 2.5 Quality assessment

The quality of all studies included was assessed by two investigators (XC and AF) independently using the Newcastle–Ottawa Quality Scale (NOS) (25) for cohort studies and the Agency for Healthcare Research and Quality (AHRQ) (26) checklist for cross-sectional studies. The NOS scale is a validated scale for non-randomized studies in meta-analysis and evaluates the risk of bias across three main domains: (1) the selection of the study groups; (2) the comparability of the groups; and (3) the ascertainment of either exposure or outcome of interest. An NOS score of 7 or more is considered high quality. The AHRQ checklist included 11 items scored as “yes,” “no,” or “unclear,” with a score of 1 for “yes” and 0 otherwise. An AHRQ score ≤3 indicates low quality, 4–7 indicates medium quality, and ≥8 indicates high quality. Disagreements were resolved through discussion with a third investigator.

### 2.6 Statistical analyses

In the present meta-analysis, the main outcome was AF occurrence risk in sarcopenic individuals during the follow-up period. The impact of sarcopenia on AF occurrence was measured by the pooled HR and 95% CI using the random-effects model with DerSimonian–Laird method (27). All eligible studies reported HRs and 95% CI of sarcopenic individuals versus the reference group (non-sarcopenic individuals), estimated from the adjusted Cox proportional hazards models. For studies that reported several multivariable-adjusted HRs comparing sarcopenia individuals versus their non-sarcopenic peers, we used the most fully adjusted estimate for potential confounders in our meta-analysis. For those only reporting ORs or RRs, we combined them with HRs. Heterogeneity between studies was evaluated using *I*^2^ statistic and Cochran's *Q* statistic, and *P* value of *Q* statistic ≤0.1 or *I*^2^ ≥ 50% was defined as significant heterogeneity (22). Moreover, a 95% prediction interval was calculated to evaluate how the true effect varied across populations (28). Preplanned subgroup analyses were conducted based on sex (men vs. women), geographic regions (Asia vs. others), age group (younger vs. older), duration of follow-up (<10 vs. ≥10 years), body mass index (BMI) category (non-obese vs. obese), and regular physical activity (yes vs. no). Meta-regression was used to determine the effect of sample size, participant average age, proportion of females, and average follow-up time.

Furthermore, sensitivity analyses were conducted by omitting one study each in turn to evaluate whether the results were affected significantly. Potential publication bias was evaluated using funnel plots and Egger's and Begg's rank tests if ≥10 studies were available (29, 30). All statistical analyses were performed using R version 4.2.2 software (31). Two-side *P*-values <0.05 were considered statistically significant. All analyses were based on previously published studies, so no ethical approval and patient consent were required.

## 3 Results

### 3.1 Research results and study characteristics

**Figure 1:**
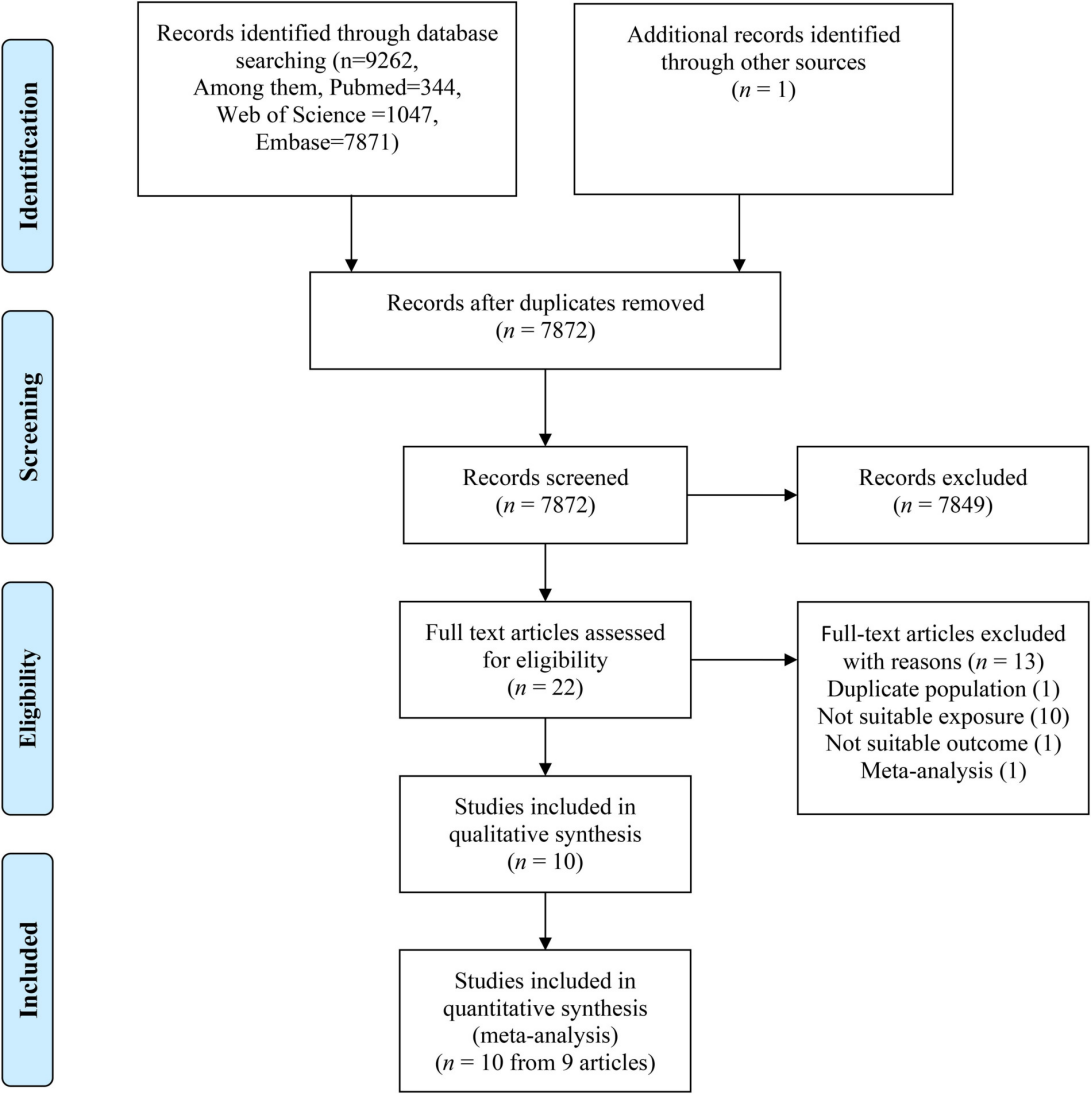
The flow diagram indicates the process of study selection.

The search initially identified 9,262 citations, of which 22 articles underwent full-text review. Among them, 13 articles did not meet the inclusion criteria, one study involved duplicate populations, one study was a meta-analysis, and 11 studies lacked suitable outcome or exposure variables. Ultimately, nine articles comprising 10 studies and 7,358,442 individuals were included in the present systematic review and meta-analysis (Figure 1).

Study characteristics are summarized in Table 1. Of these, nine studies were prospective cohort studies, and one study was cross-sectional. Six studies were conducted in Asia and four in Europe. The studies used a variety of sarcopenia criteria, including both muscle mass and muscle function (*n* = 3), single muscle mass (*n* = 4), and single muscle function (*n* = 3). Muscle mass assessments were frequently measured by DXA (*n* = 5), followed by BIA (*n* = 2). All studies used hand grip strength (HGS) for muscle function assessment. In addition, five studies used AWGS criteria and two used EWGSOP criteria to diagnose sarcopenia. The sample size of the included studies ranged from 827 to 2,673,108, with a mean study duration varying from 2 to 26.3 years. The mean age of the participants ranged from 18.3 to 76 years. All included studies adjusted for multiple covariates, including age, sex, and other risk factors.

**Table 1:** Characteristics of nine articles with 10 observational studies included in the present meta-analysis.

Authors, Year	Study design	Country	Sample size	Source of cohort	Mean age	Mean follow up	Proportion of female (%)	Measurement muscle mass	Measurement muscle function	Definition source	component of sarcopenia criteria	Outcome type	Reference group
Yu et al. 2025	Community-dwelling prospective cohort study	China	4,321	non-diabetic participants aged ≥60	68.05	10.9	46.73	DXA	Dynamometer	ESPEN	Muscle mass + HGS	incidence	Non-sarcopenia
Tang et al. 2024	Prospective cohort study	UK	384,433	general Caucasian participants	58	12.56	54.3	BIA	Dynamometer	EWGSOP2	Muscle mass + HGS	incidence	Non-sarcopenia
Shim et al. 2024	Prospective cohort study	Korea	2225	general participants	76	2	54.2	DXA	Dynamometer	AWGS	Muscle mass + HGS	incidence	Non-sarcopenia
Xia et al. 2021	Community-dwelling cross-sectional study	China	2,432	middle-aged and elderly participants	62.2	—	59.21	DXA	NA	AWGS	muscle mass	prevalence	Non-sarcopenia
Woo et al. 2023 (men and women)	Prospective cohort study	Korea	26,73,108	general participants without AF	48.58	9.5	48.6	DXA	NA	AWGS	Muscle mass	incidence	Lowest quintile of ASMMI
Liu et al. 2025	Community-dwelling prospective cohort study	China	8,060	non-diabetic individuals aged ≥60	68	9.7	50.2	BIA	NA	AWGS	Muscle mass	incidence	Normal ASMMI group
He et al. 2024	Prospective cohort study	UK	487,673	general participants	56.4	12.4	54.8	NA	Dynamometer	EWGSOP2	HGS	incidence	Low HGS
Kunutsor et al. 2020	Population-based prospective cohort study	Finland	827	general participants aged 61 to 74 years without AF	69	15.7	54	NA	Dynamometer	Lowest tertile of HGS sarcopenia	HGS	incidence	Lowest tertile of HGS
Andersen et al. 2015	Population-based prospective cohort study	Sweden	11,22,255	general young male participants	18.3	26.3		NA	Dynamometer	lowest quintile of HGS as sarcopenia	HGS	incidence	Lowest quintile of HGS

DXA, dual-energy X-ray absorptiometry; BIA, bioelectrical impedance analysis; ESPEN, European Society for Clinical Nutrition and Metabolism; EWGSOP, European Working Group on Sarcopenia in Older People; AWGS, Asian Working Group for Sarcopenia; HGS, hand grip strength; ASMMI, appendicular skeletal muscle mass index.

### 3.2 Quality of study

The risk of bias was assessed using the NOS for prospective cohort studies and the AHRQ checklist for cross-sectional study. Among the prospective cohort studies, all studies were rated as high quality with NOS score ≥7. The cross-sectional study received a quality score of 8, denoting high quality. More detailed information on the quality assessment of the included studies is provided in Supplementary Tables 1 and 2.

### 3.3 Sarcopenia and AF

In the random-effects meta-analysis of 10 cohorts studies, sarcopenia was significantly associated with increased risk of AF compared with non-sarcopenic individuals (reference group), and the pooled HR for AF incidence risk was 1.44 (95% CI: 1.22–1.68, *P* = 0.001); however, heterogeneity assessment using *χ*^2^ (0.04) and *I*^2^ (92.29%) tests indicated high heterogeneity between studies. Furthermore, the prediction interval was 95% predictive interval (PI) [0.91–2.26]. This range is typically wider than the confidence interval, particularly in the presence of high heterogeneity, as observed in our meta-analysis. The PI, together with the significant heterogeneity, underscores the need for further investigation into potential moderating factors influencing the average effect sizes.

**Figure 2:**
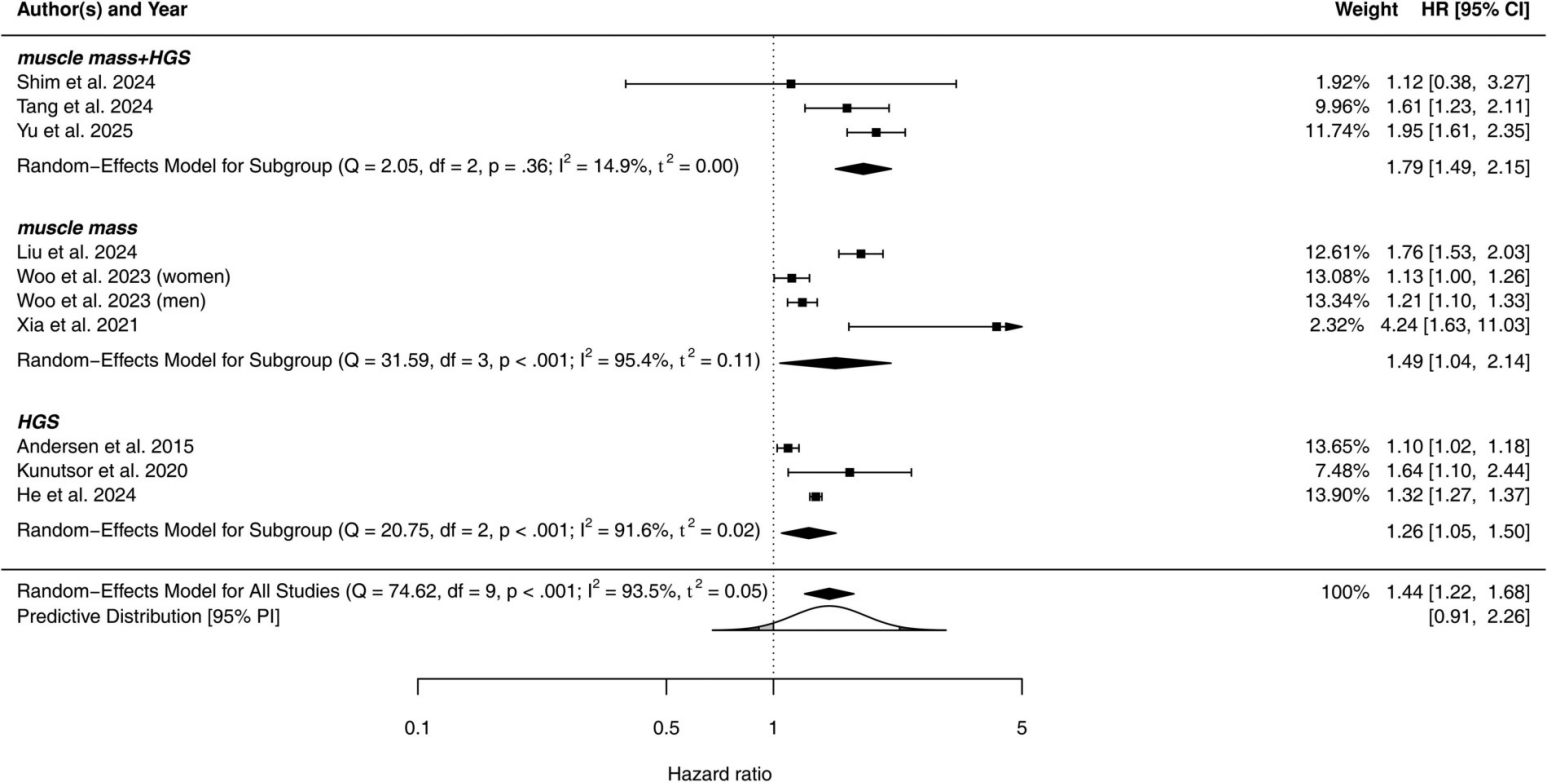
Forest plot analysis for overall effect of sarcopenia on atrial fibrillation, and respective effect according components of sarcopenia definition. HGS, hand grip strength; HR, hazard ratio; PI, predictive interval.

The analysis according to different sarcopenia criteria (combined or single components of muscle mass and function) is shown in Figure 2. When strict criteria were used, the effect of sarcopenia remained positively significantly associated with AF risk, with a 79% higher risk among sarcopenic individuals compared with non-sarcopenic peers (pooled HR: 1.79, 95% CI: 1.24–2.15, *P* < 0.001). Heterogeneity within this subgroup was low and no longer significant (*I*^2^ = 14.9, *P* = 0.36). Similarly, in the four studies using muscle mass and three studies using HGS only, sarcopenia was consistently associated with an increased risk of AF, but the association strengths was attenuated, with pooled HRs of 1.49 (95% 1.04–2.14, *P* = 0.029) and 1.26 (95% 1.05–1.37, *P* = 0.01), respectively. Heterogeneity within these two subgroups remained high (*I*^2^ = 95.4% and 91.6%), potentially due to varying cutoff values.

### 3.4 Subgroup analyses

Sarcopenia was consistently associated with a higher risk of AF onset across all subgroups analyzed (Table 2). In particular, the results indicated that subjects with sarcopenia—compared with those without—had a significantly increased risk of AF onset irrespective of sex. However, there was a higher risk in women (HR = 1.97, 95% CI: 1.17–3.31) compared with men (HR = 1.44, 95% CI: 1.19–1.74). This pattern (sarcopenia vs. non-sarcopenia) was also found in both age groups with pooled adjusted HRs of 1.22 (95% CI: 1.11–1.34) and 1.69 (95% CI: 1.42–2.01) in the younger and older age groups, respectively. Similarly, sarcopenia was associated with increased AF risk in non-obese and obese subjects (pooled adjusted HR: 1.37, 95% CI: 1.11–1.71 and 1.86, 95% CI: 1.19–2.91, respectively). The association persisted among those with regular and irregular physical activity (pooled adjusted HR: 1.5, 95% CI: 1.08–2.08 and 1.46, 95% CI: 1.37–1.54, respectively). In addition, there was no evidence of geographic influence, as pooled HRs remained significant in both Asian cohorts (HR: 1.54, 95% CI: 1.17–2.02) and non-Asian cohorts (HR: 1.33, 95% CI: 1.16–1.52). Patients with sarcopenia had a >1.3-fold greater risk of AF onset according to adjusted analyses regardless of duration of follow-up (≥10 years or <10 years).

**Table 2:** Subgroup analyses between sarcopenia and risk of atrial fibrillation by sex, age, BMI category, regular physical activity, duration of follow-up, and geographic location.

Subgroup	No. of studies	Adjusted HR (95% CI)	*I*^2^ (%)	*P*_heterogeneity_
Sex				
Men	4	1.44 (1.19, 1.74)	69.1	0.024
Women	4	1.97 (1.17, 3.31)	92.86	<0.001
Age				
Younger	5	1.22 (1.11, 1.34)	82.79	<0.001
Older	6	1.69 (1.42, 2.01)	62.39	0.009
BMI				
Non-obese	5	1.37 (1.11, 1.71)	91.69	<0.001
Obese	6	1.86 (1.19, 2.91)	98.73	<0.001
Regular physical activity				
Yes	3	1.50 (1.08, 2.08)	74.58	0.016
No	3	1.46 (1.37, 1.54)	0	0.664
Duration				
<10 years	5	1.34 (1.11, 1.61)	87.06	<0.001
≥10 years	5	1.46 (1.18, 1.80)	94.61	<0.001
Region				
Asian	6	1.54 (1.17, 2.02)	92.17	<0.001
Non-Asian	5	1.33 (1.16, 1.52)	86.98	<0.001

HR, hazard ratio; BMI, body mass index.

### 3.5 Meta-regression analyses

Meta-regression analyses showed no association between pooled adjusted HRs and either the proportion of females (*P* = 0.482) or average follow-up time (*P* = 0.387). However, there was a significant positive relationship with participants' average age (Coef.: 0.010, 95% CI: 0.004–0.017, *P* = 0.002) and a negative relationship with sample size (Coef.: −2 × 10^−7^, 95% CI: −3 × 10^−7^–0; *P* = 0.005) (Supplementary Table 3).

### 3.6 Sensitivity analyses and publication bias

**Figure 3:**
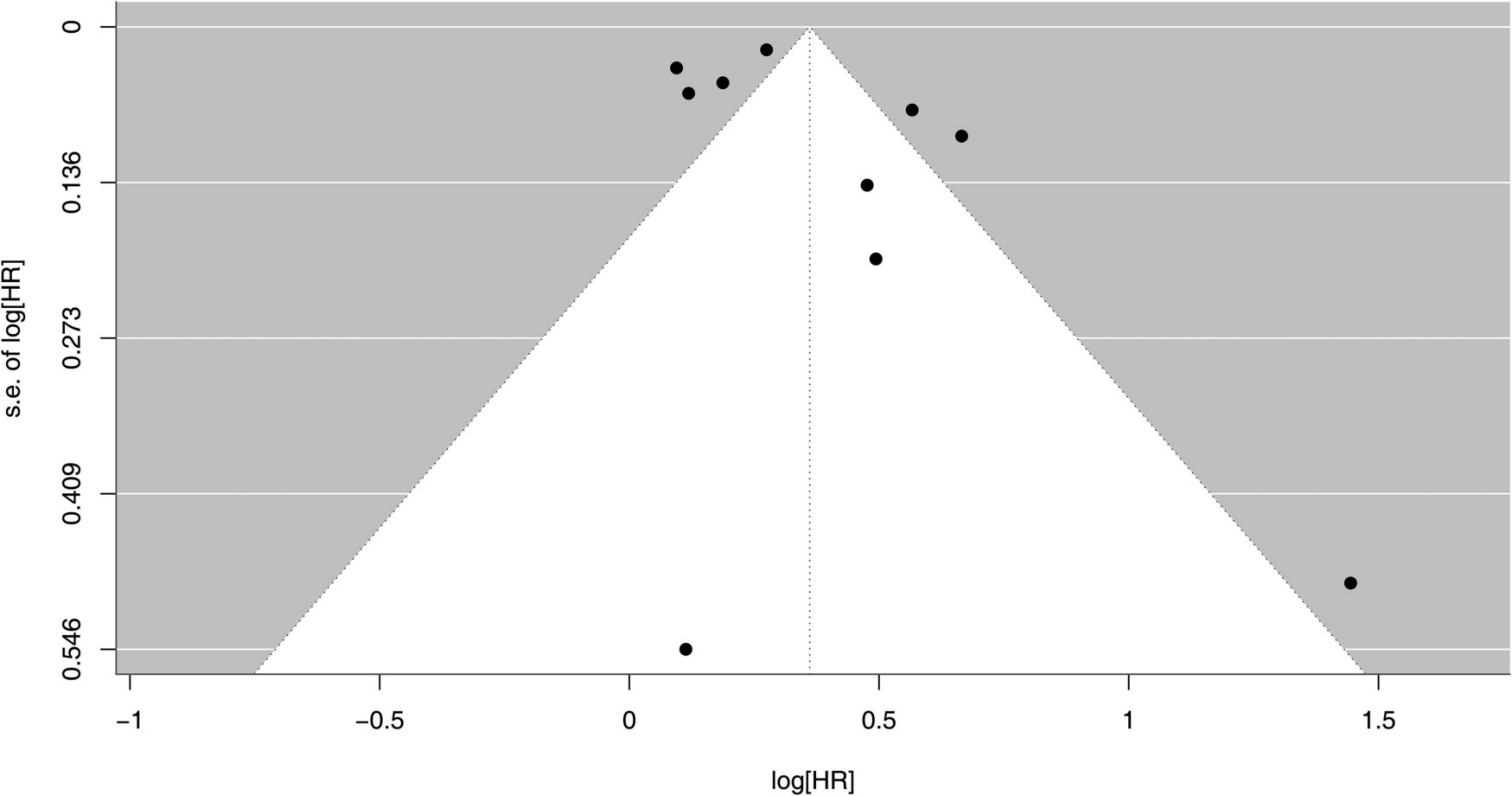
Funnel plots of sarcopenia effect on atrial fibrillation from studies. HR, hazard ratio; s.e., standard error.

Sensitivity analyses were further conducted to examine the stability of the results. After removing one study at a time, a consistent significant association between sarcopenia and increased risk for AF incidence remained [HR with 95% CI ranging from 1.36 (1.20–1.54) to 1.47 (1.28–1.69)], indicating robustness of the meta-analysis results. Moreover, the funnel plot for the outcome revealed symmetry, implying no evidence of publication bias in this meta-analysis (Figure 3). This was further justified by both Begg's test and Egger's regression test, with *P* values of 0.728 and 0.278, respectively.

## 4 Discussion

In this systematic review and meta-analysis of 10 studies involving 735,844 individuals, those with sarcopenia had 1.44-fold increased risk of AF incidence compared with their non- sarcopenia peers. This robust association was further supported across both single and combined sarcopenia criteria, sensitivity analyses, and absence of publication bias. Moreover, subgroups analyses were conducted to assess whether sex, age, duration of follow-up, geographical location, BMI, and physical activity influenced the association. All secondary analyses were significant, expect the aspect of physical activity, with HRs ranging from 1.22 to 1.97; in particular, there was a significant difference in HR estimates between the sexes (men and women).

While the pooled HR for risk of AF onset was consistently significant across almost all subgroups, the HR value was as much as 1.26 when single component of muscle strength (i.e., HGS) was used for sarcopenia diagnosis, whereas it raised to 1.79 when combined use of both muscle mass and function. These results confirm that patients who met the comprehensive definition of sarcopenia were prone to more frailty in both muscle mass and function, thus leading to an accumulated risk of AF incidence. Few studies concurrently analyzed the effect of muscle mass and function on AF risk. Tang et al. reported an inverse association of both muscle mass and strength with risk of AF onset, with muscle mass exerting a greater effect than muscle strength (18). Similarly, the UK Biobank cohort reported that lower HGS was significantly associated with 1.71-fold and 1.42-fold increases in CVD mortality in men and women, respectively, but this risk was 3.08-fold and 1.85-fold increased with lower muscle mass in men and women (32). Low HGS is considered a precursor of sarcopenia (33) and is recognized by the AWGS as “possible sarcopenia,” warranting early intervention in primary health care and preventive services (34). Indeed, prior intervention studies suggested that both aerobic and resistance exercise can effectively maintain muscle function and enhance muscle mass in elderly populations with presarcopenia, thereby reducing AF development and other CVD morbidities (35, 36). Heart failure and AF are closely related, with each condition promoting and worsening the other. They share common risk factors—such as age, metabolic disorders, and decreased skeletal muscle quality and strength (i.e., sarcopenia)—and frequently coexist, leading to higher rates of mortality. Indeed, many studies have reported a remarkable prevalence of sarcopenia in patients with heart failure, particularly in the acute decompensated heart failure subtype (37). For example, a cohort study of 400 patients with normal perfusion and preserved left ventricular ejection fraction demonstrated that lower skeletal mass index (SMI), an important indicator of sarcopenia, was independently associated with a 1.87-fold higher risk of heart failure hospitalization for every 10 cm^2^/m^2^ decrease in SMI. This risk was particularly observed in young, female, and obese patients (38).

It is noteworthy that sarcopenic women appeared to be more affected with AF according to our pooled analysis. This sex-specific difference is prominent in the aspects of AF epidemiology, pathophysiology, clinical presentation, and prognosis (39), but findings are still inconclusive regarding AF susceptibility in sarcopenia (40, 41). Some studies demonstrated a higher incidence of AF in men (18, 40), but some reported contrasting findings (8). For example, pooled analysis from the BiomarCaRE Consortium showed that the cumulative risk of developing AF was higher in men than in women across most of the lifespan; the study also highlighted an important attributed effect of obesity to sex discrepancies (40). Similarly, the VITAL trial—a large contemporary, prospective cohort—found that women were at lower risk for incident AF than men after adjusting for BMI and other confounders; but intriguingly, this association was reversed with an increased risk of AF in women after adjusting for height and weight or body surface area (41). In the present meta-analysis, our data reported an increased AF risk in sarcopenic women compared with men, consistent with findings from a recent Chinese prospective study of 384,433 participants, which reported a nearly 4-fold higher AF incidence in women with sarcopenia compared with their counterparts without sarcopenia after a 12-year follow-up. This related risk was attenuated in the male subpopulation (18). Another meta-analysis of 263 studies involving 692,056 individuals found a higher prevalence of sarcopenia in women than men, according to the International Working Group on Sarcopenia (17% vs. 12%) (8). Other studies speculated that the predisposition to CVDs such as AF in sarcopenic women was attributed to several risk factors, including elevated insulin resistance (IR) and abnormal sex hormone disturbance. As such, despite limited evidence, the present study provides insights into this area and highlights the need for further research into sex-related mechanisms of AF incidence in sarcopenia.

Notably, our meta-analysis revealed that obese individuals with sarcopenia had a more elevated risk of AF onset. This finding, which is consistent with several related works (42, 43), emphasizes the clinical significance of obesity and abdominal distribution of fatty tissue in patients with AF (42). In a community-based study of middle-aged and elderly Chinese participants, Xia et al. reported that the prevalence of AF in people with sarcopenic obesity (SO) is 7.5%, significantly higher than their non-sarcopenic or lean counterparts. Importantly, the significant association between sarcopenia and AF was only observed for overweight/obese, not in sarcopenic normal-weight participants (43). Consistent findings were provided by another prospective cohort that reported that the risk of long-term AF in non-diabetic elderly was 2.7- and 2-fold higher in those with SO and sarcopenia alone, respectively (20). Sarcopenia and obesity are two well-recognized comorbidities and they might interact to affect function of the cardiac conduction. As reported previously, sarcopenia is associated with impaired left ventricular diastolic dysfunction (44), consequently leading to profibrillatory remodeling of the left atrium and ultimately contributing to AF development (45). Meanwhile, loss of skeletal muscle mass—a major characteristic of sarcopenia—is always accompanied by adipose tissue deposition (46–48). Excess pericardial fat accumulation in obesity can lead to the fatty infiltration of the myocardium and atrial septum (49). Moreover, sarcopenia and obesity exert a synergistic effect on IR and inflammation (50, 51), which promote an increased risk AF onset as they act as crucial mediators or collaborators. Indeed, this hypothesis was confirmed by Yu and colleagues, showing that estimated glucose disposal rate, hsCRP, and galectin-3—surrogate markers of IR and inflammatory reaction—accounted for more than 80% of the association between SO and AF risk (20). To some extent, obesity contributes to both high lean body mass and fat body mass. Fenger-Grøn and colleagues reported that lean body mass was the predominant anthropometric risk factor for AF (52). High lean body mass may coexist with loss of muscle mass, as seen in sarcopenic obesity, and both factors are associated with higher risk of AF, as demonstrated by the Mendelian randomization study (53) and other cohort studies (54, 55). These findings highlight the need for special attention to sarcopenic individuals with obesity or lean body mass for effective prevention of AF.

In addition, the present meta-analysis revealed that regular physical activity was not a favorable factor in reducing AF onset among people with sarcopenia. These unexpected findings are consistent with previous studies (56, 57). For example, Andersen et al. demonstrated that moderate-intensity exercise was associated with a reduced risk of AF, whereas long-term high-intensity endurance exercise increased AF risk (57). Similarly, Elliott and colleagues (58) found a 12% increase in incident AF associated with a high dose of vigorous physical activity, consistent with data from the Tromsø Study (59). In contrast, data from the UK Biobank reported a significant inverse association between higher levels of physical activity and AF, including a 60% lower risk for AF among individuals with high genetic AF risk (60). Likewise, data from the Rotterdam Study found no association between total physical activity or any specific activity type and AF risk in older adults after 16.8 years of follow-up (61). These discrepancies may be multifactorial and explained by differences in the confounders used, age distribution, physical activity assessment, and sex. Two recent meta-analyses that included 16 and 23 prospective studies reported sex-specific differences in association of physical activity with AF, with more pronounced reduced risk in women, but not in men (62). Extending this evidence, Kunutor and colleague found a null association between regular physical activity and AF risk in the general population, but the results suggest a sex-specific divergence in the associations—an increased risk in men and a decreased risk in women (63). In the present meta-analysis, the number of included studies was limited in both regular and irregular physical activity categories; therefore, conclusions regarding AF risk among those with sarcopenia engaging in physical activity should be interpreted with caution, and further evaluation is warranted to fully clarify the contribution of physical activity to AF risk.

Emerging literature has highlighted many secondary causes of AF independent of sarcopenia, covering metabolic disorders (64), lifestyle factors (65, 66), physical exercise types and intensities (67), cardiac channelopathies (68), and cardiomyopathy (69). Some studies showed that AF can be precipitated by hypertension and hyperthyroidism, particularly in younger subgroups (64, 70). In a Korean nationwide cohort that included 2,958,544 subjects aged 20–39 years without antihypertensive medication use, Lee and colleagues reported that two-fifths of young adults had hypertension after a median follow-up of 8.3 years. Moreover, isolated diastolic, isolated systolic, and systolic–diastolic hypertensions were consistently associated with higher risks of incident AF when compared with normal blood pressure (70). Similarly, another cohort study confirmed that prehypertension and hypertension had a more prominent contributory role in the development of incident AF in a younger age group than in an older age group (71). Subclinical hyperthyroidism and high-normal free T4 are also well-established risk factors for AF onset. Hyperthyroidism-related AF is reversible, with most patients spontaneously reverting to sinus rhythm within 4–6 months during or after restoration of euthyroidism. Therefore, restoring thyroid function is an indispensable element in hyperthyroidism-related AF management (72). For example, in an early population-based study of 40,628 Danish patients with hyperthyroidism, Frost and colleagues found the risk of AF to be higher in men than in women, and this risk progressed with age across the age range of 20–89 years. Consistently, a pharmacoepidemiologic cohort study reported that treatment of subclinical hyperthyroidism was associated with a 75% lower risk of AF compared with untreated counterparts after a 4-year follow-up (73).

Lifestyle factors particularly pertinent to young patients include alcohol consumption, smoking, and endurance exercise (66, 74). Long-term habitual alcohol intake, which is very common in young people, has been shown to increase the risk of AF by 34% (66), with this risk increasing as much as 47% among persistent heavy drinkers aged 20–39 years (75). With regard to smoking, Chamberlain and colleague reported a 2-fold increased risk of AF attributed to current smoking (65). Consistently, Aune and colleagues using a meta-analysis reported that the risk for AF was 1.32 and 1.21, 1.09 times higher for current, ever and former smokers compared to never smokers, implying a dose–response manner (76). These findings suggest that changes in alcohol consumption and smoking habits could be beneficial for AF prevention (77). Moreover, endurance sport has been implicated as a detrimental factor for AF (67, 78). The Tromsø Study revealed that both prevalence and adjusted risk of AF were consistently higher in athletes compared with non-athletes over 14 years of endurance sport practice (79). A meta-analysis supported this evidence, showing that the risk of developing AF had significantly doubled in athletes compared with non-athlete controls, especially among younger individuals practicing endurance sport (80).

Cardiomyopathy is another important cause of AF and a strong risk factor for thromboembolic events, progression to heart failure, and mortality or heart transplantation (68). In a meta-analysis that included 220 studies with 118,668 participants with cardiomyopathies, the prevalence of AF was nearly 20% in participants with dilated, ischemic, and hypertrophic cardiomyopathies, but only 5% in patients with peripartum cardiomyopathies, regardless of sex (81). Likewise, real-world data including 634,885 patients with cardiomyopathy, in a study conducted by Buckley and colleagues, revealed that AF was significantly associated with a 1.3 times higher mortality risk for patients with hypertrophic and dilated cardiomyopathy. Moreover, higher risks of hospitalization, incident heart failure, and stroke were consistently found in all cardiomyopathy subtypes with concomitant AF (68). Similar findings were confirmed by another Chinese study (82), in which AF was highly prevalent in cardiomyopathy, and its presence was positively associated with the risk of stroke and heart failure, with a 36% higher risk compared with those without AF. Furthermore, cardiac channelopathies are also recognized as relevant cause of AF, and some subtypes should be suspected particularly in young patients with early AF onset (69, 83). Cardiac channelopathies are a group of clinical syndromes caused by mutations in genes encoding specific cardiac ionic channels; these ion channel dysfunctions are found highly present in AF, which often may worsen these conditions as they are associated with more adverse clinical outcomes. Notably, major inherited channelopathies included Brugada syndrome (BrS), long QT syndrome (LQTS), short QT syndrome (SQTS), and early repolarization syndrome (84). The epidemiology, pathophysiology, risk factor, and management of AF for cardiac channelopathies could vary according to their subtypes. For example, the prevalence of AF is reported to be as high as 70% and 53% in SQTS and BrS, respectively, whereas it was nearly 30% in LQTS (84). Within LQTS subtype, a large population-based study revealed a J-shaped relationship of QTc duration with the risk of AF after a 5.7-year follow-up (85). Johnson and colleagues identified LQT1 and the male sex as risk factors, with higher prevalence of AF in the LQT1 subtype and male subgroups when compared with other genetic subtypes and females, respectively (86). Beta-blocker therapy is considered the standard first-line therapy for LQTS patients, with potentially more benefit for LQTS1 patients (69). In BrS, AF is often under-detected with routine methods; therefore, the presence of AF could lead to a more malignant course, with a greater incidence of syncope and ventricular fibrillation. For example, a prospective study of 51 patients with BrS revealed that nearly 14% of inappropriate shocks were caused by atrial arrhythmias (87). Collectively, AF may be the first manifestation of an undiagnosed channelopathy, and proper diagnosis carries clinical prognostic implications due to the increased risk of sudden cardiac death.

There is growing interest in identifying the mechanisms underlying the strong association between sarcopenia and AF. Sarcopenia is involved in the pathophysiological processes of skeletal muscles, and it contributes to induce oxidative stress injury in cardiomyocytes by stimulating mitochondrial dysfunction, chronic inflammation, and overproduction of reactive oxygen species, which worsen lipotoxic damage on cardiomyocytes (88, 89). This might explain the deleterious effect of sarcopenia on AF risk. Furthermore, a progressive accumulation of fibrosis in the cardiac conduction system in patients with sarcopenia has been observed (90), with increased atrial fibrosis responsible for accelerating atrial remodeling that could further promote AF (91). Finally, sarcopenia is also an established risk factor of impaired left ventricular diastolic dysfunction (92), and the latter might lead to induce AF by overloading left atrial, and remodeling of the left atrium. For example, HGS, an important diagnostic indicator of the sarcopenia, is associated with a series of cardiac structures and functions, and its significant deficiency is linked to cardiac hypertrophy and remodeling (93, 94). Further research is required to elucidate the mechanisms underlying the role of sarcopenia in onset of AF.

### 4.1 Limitations

We acknowledge the following limitations. First, despite the high quality of the included prospective cohort studies, the potential of unmeasured confounding might introduce bias and influence the study results due to nature of the observational design. Second, although the HRs were extracted from the fully adjusted Cox regression models, the confounding factors varied across studies. Third, the inclusion of studies that used non-comprehensive criteria for sarcopenia due to limited availability of data with comprehensive criteria may cause bias. We performed subgroup analyses to overcome these limitations. Finally, substantial heterogeneity was detected in our meta-analysis and could not be fully explained by subgroup stratification, but sensitivity analyses indicated that results remained consistent after removing one study each time.

## 5 Conclusions

In conclusion, the present meta-analysis of prospective cohort studies showed that sarcopenia in adults was significantly associated with a 1.42-fold higher risk of AF. Consistent findings were observed across subgroups defined by sex, age, geographical region, durations of follow-up, BMI, and physical activity levels. These findings highlight the importance of early muscle assessment—including muscle mass, strength, and function—in routine clinical practice. Regular monitoring for sarcopenia may maximize opportunities to reduce their risk of AF onset and other comorbidities.

## Data Availability

The original contributions presented in the study are included in the article/Supplementary Material, further inquiries can be directed to the corresponding authors.
