# Supplementary material for: Effect of sarcopenia on risk of atrial fibrillation: a systematic review and meta-analysis of observational studies
Source: Front Cardiovasc Med. 2026 Apr 1;13:1721219. doi: 10.3389/fcvm.2026.1721219 (PMC13079711; doi:10.3389/fcvm.2026.1721219)
Supplement: Supplementary file 1 [file Datasheet1.docx]

Supplementary Material

Supplementary Table 1. Quality assessment of observational studies using Newcastle–Ottawa Scale.

| **Authors** | **Study design** | **Exposed representation** | **Selection of the non-exposed cohort** | **Ascertainment of exposure** | **outcome of interest was not present at start of study** | **Comparability of cohorts** | **Assessment of outcome** | **Sufficient follow-up time for outcomes to occur** | **Adequacy of follow up of cohorts** | **Total Points** |
| --- | --- | --- | --- | --- | --- | --- | --- | --- | --- | --- |
| Yu et al. 2025 | prospective cohort | 0 | 1 | 1 | 1 | 2 | 1 | 1 | 1 | 8 |
| Tang et al. 2024 | prospective cohort | 1 | 1 | 1 | 1 | 2 | 1 | 1 | 1 | 9 |
| Shim et al. 2024 | prospective cohort | 1 | 1 | 1 | 1 | 2 | 1 | 0 | 1 | 8 |
| Liu et al. 2024 | prospective cohort | 0 | 1 | 1 | 1 | 2 | 1 | 1 | 1 | 8 |
| Ye et al. 2024 (some UK Bio with Tang2024) | prospective cohort | 1 | 1 | 1 | 1 | 2 | 1 | 1 | 1 | 9 |
| Kunutsor et al. 2020 | prospective cohort | 1 | 1 | 1 | 1 | 2 | 0 | 1 | 1 | 8 |
| Andersen et al. 2015 | prospective cohort | 1 | 1 | 1 | 1 | 2 | 1 | 1 | 1 | 9 |
| Woo et al. 2023 | prospective cohort | 1 | 1 | 1 | 1 | 2 | 1 | 1 | 1 | 9 |
| Trevisan et al. 2016 | prospective cohort | 1 | 1 | 1 | 1 | 1 | 1 | 1 | 0 | 7 |

Supplementary Table 2. Quality assessment of observational studies using the Agency for Healthcare Research and Quality.

|  | 1) Define the source of information (survey, record review) | 2) List inclusion and exclusion criteria for exposed and unexposed subjects (cases and controls) or refer to previous publications | 3) Indicate time period used for identifying patients | 4) Indicate whether or not subjects were consecutive if not population-based | 5) Indicate if evaluators of subjective components of study were masked to other aspects of the status of the participants | 6) Describe any assessments undertaken for quality assurance purposes (e.g., test/retest of primary outcome measurements) | 7) Explain any patient exclusions from analysis | 8) Describe how confounding was assessed and/or controlled. | 9) If applicable, explain how missing data were handled in the analysis | 10) Summarize patient response rates and completeness of data collection | 11) Clarify what follow-up, if any, was expected and the percentage of patients for which incomplete data or follow-up was obtained |
| --- | --- | --- | --- | --- | --- | --- | --- | --- | --- | --- | --- |
| Xia et al. 2021 | Yes | Yes | Yes | Yes | Yes | Unclear | Yes | Yes | No | Yes | No |

Supplementary Table 3. Meta-regression of relative risk of mortality among sarcopenia vs. non-sarcopenia groups.

| Variable | Coefficient | SE | Z value | P value | CI-Lower | CI-Upper |
| --- | --- | --- | --- | --- | --- | --- |
| Participants’ average age | 0.010 | 0.003 | 3.132 | 0.002 | 0.004 | 0.017 |
| Sample size | -2$\times$10-7 | 0 | -2.799 | 0.005 | -3$\times$10-7 | 0 |
| Proportion of female | 0.02 | 0.028 | 0.704 | 0.482 | -0.036 | 0.076 |
| Average follow-up time | -0.012 | 0.014 | -0.865 | 0.387 | -0.032 | 0.015 |
